# Amniotic fluid-derived multipotent stromal cells drive diabetic wound healing through modulation of macrophages

**DOI:** 10.1186/s12967-020-02674-5

**Published:** 2021-01-06

**Authors:** Bibi S. Subhan, Jennifer Kwong, Joseph F. Kuhn, Arie Monas, Sonali Sharma, Piul S. Rabbani

**Affiliations:** Hansjörg Wyss Department of Plastic Surgery, New York University School of Medicine, 540 First Avenue, New York, 10016 USA

**Keywords:** Amniotic fluid multipotent stromal cells, Diabetic wounds, Cellular therapy

## Abstract

**Background:**

Cutaneous wounds in patients with diabetes exhibit impaired healing due to physiological impediments and conventional care options are severely limited. Multipotent stromal cells (MSCs) have been touted as a powerful new therapy for diabetic tissue repair owing to their trophic activity and low immunogenicity. However, variations in sources and access are limiting factors for broader adaptation and study of MSC-based therapies. Amniotic fluid presents a relatively unexplored source of MSCs and one with wide availability. Here, we investigate the potential of amniotic fluid-derived multipotent stromal cells (AFMSCs) to restore molecular integrity to diabetic wounds, amend pathology and promote wound healing.

**Method:**

We obtained third trimester amniotic fluid from term cesarean delivery and isolated and expanded MSCs in vitro. We then generated 10 mm wounds in Lepr^db/db^ diabetic mouse skin, and splinted them open to allow for humanized wound modeling. Immediately after wounding, we applied AFMSCs topically to the sites of injuries on diabetic mice, while media application only, defined as vehicle, served as controls. Post-treatment, we compared healing time and molecular and cellular events of AFMSC-treated, vehicle-treated, untreated diabetic, and non-diabetic wounds. A priori statistical analyses measures determined significance of the data.

**Result:**

Average time to wound closure was approximately 19 days in AFMSC-treated diabetic wounds. This was significantly lower than the vehicle-treated diabetic wounds, which required on average 27.5 days to heal (p < 0.01), and most similar to time of closure in wild type untreated wounds (an average of around 18 days). In addition, AFMSC treatment induced changes in the profiles of macrophage polarizing cytokines, resulting in a change in macrophage composition in the diabetic wound bed. We found no evidence of AFMSC engraftment or biotherapy induced immune response.

**Conclusion:**

Treatment of diabetic wounds using amniotic fluid-derived MSCs encourages cutaneous tissue repair through affecting inflammatory cell behavior in the wound site. Since vehicle-treated diabetic wounds did not demonstrate accelerated healing, we determined that AFMSCs were therapeutic through their paracrine activities. Future studies should be aimed towards validating our observations through further examination of the paracrine potential of AFMSCs. In addition, investigations concerning safety and efficacy of this therapy in clinical trials should be pursued.

## Background

The urgent need for effective treatment of chronic diabetic ulcers has seen a surge in utilization of mesenchymal cells with primitive stem-like properties. Diabetes and associated complications affect 9.4% of the population of the United States, or 1 in every 11 individuals [1]. Chronic non-healing ulcers are one of the most conspicuous manifestations of this disease, costing an estimated $1.38 billion annually [2]. This monetary value, however, does not account for psychosocial and economic impacts on the quality of life for both patients and caregivers. Currently, most available healthcare options address wound management, but not therapy of these diabetic wounds. The search for innovative and effective solutions that can target the complex pathology of diabetic ulcers has recently uncovered the potential of mesenchymal progenitor cells.

Several studies have explored the capability of mesenchymal progenitor cells derived from adipose tissue, bone marrow, umbilical cord among other sources for tissue repair and regeneration through treatment of cardiac [3], hepatic [4], renal [5], ischemic injuries [6, 7] and cutaneous wounds [8] to name a few. In our previous work, we have demonstrated the use of bone marrow-derived multipotent stromal cells for effectively promoting closure of mouse type II diabetic wounds [9]. Among the potential cell types being considered for therapy, an under-utilized source is human amniotic fluid-derived multipotent stromal cells (AFMSCs). Amniotic fluid, composed mainly of water, also contains a fluctuating solute content of essential nutrients and immune effectors, and a population of cells that upon adherent culture enrich for mesenchymal progenitors [10]. This fluid is clinically utilized for pre-natal analysis and diagnostics, then typically discarded at childbirth, but is in fact an abundant and widely available source of mesenchymal progenitor cells with remedial potential.

A liter of amniotic fluid contains approximately 7 million mononucleated cells [11], while ~ 5 × 10^5^ cells per liter demonstrate plastic adherence in cell culture conditions [12]. These amniotic fluid cells have easy expansion in readily available standard reagents [10, 13], and in just 4 weeks of growth, 180 million cells with AFMSC morphology can be derived from 2 mL of amniotic fluid [14]. Adverse events, such as tumor formation, due to administration of AFMSCs have not been reported according to the literature, an indication of the safety of their allogeneic use [15, 16]. Additionally, there are no ethical stipulations as for embryonic stem cells, since they are obtained from an adult source [17]. Despite their source, in vitro analysis of AFMSCs have characterized them with markers of both adult and embryonic stem cells [18, 19]. Although inherent pluripotent potential of these AFMSCs has not been unanimously established, compared to adult stem cells they do have greater proliferation and more widespread differentiation [10, 20–22].

AFMSCs are a heterogeneous population but demonstrate capacities similar to adult mesenchymal progenitor cells of reliable differentiation along mesenchymal lineages like osteogenic, adipogenic and chrondrogenic [19, 21, 23]. These cells have high proliferative capacity and demonstrate multipotency, much like adult mesenchymal multipotent cells. The cells do not require feeder layers and reliably proliferate in mesenchymal type cell culture media. Full-term and early-term AFMSCs show similar characteristics, pointing to full term amniotic fluid as an easily accessible source of therapeutic cells [13, 24].

A few studies have reported efficacy of AFMSCs in wound healing models via modulation of the inflammatory phase of healing [25, 26]. The majority of studies using AFMSCs have investigated cardiovascular, gastrointestinal, neural, respiratory and muskulo-skeletal tissue repair models [20]. Their potential for fulfilling the public health need of an effective therapy for accelerating treatment of non-healing diabetic wounds requires careful investigation. Previous molecular analyses of diabetic wounds have related deficits in key physiological elements and events including growth factor and macrophage responses, angiogenic activation, and overall cellular and extracellular matrix migration and remodeling [27]. These impairments result in an overall chronically inflamed, non-healing phenotype of the diabetic wounds. Therapy aimed at diabetic wounds should have the ability to coordinate molecular events towards normal wound healing stages.

In this study, we cultured heterogeneous mesenchymal progenitor cells from human full-term gestation amniotic fluid, and assessed the efficacy of these AFMSCs in promoting wound closure in a type II diabetic full-thickness pre-clinical wound model. We find that a single topical application of AFMSCs is sufficient to drive tissue repair, and to reduce pathologic healing time. In particular, we identified polarization of wound site macrophages towards anti-inflammatory phenotypes in response to AFMSC administration, to accelerate diabetic wound closure.

## Methods

### AFMSC culture

Collection of amniotic fluid and derived products was approved by the Institutional Review Board at New York University School of Medicine. Amniotic fluid was collected at the time of scheduled, term cesarean delivery with the patient’s informed consent (study# i15-01269, New York, NY, USA) and cultured per published protocols to enrich for AFMSCs [13]. Briefly, amniotic fluid stem cells were isolated from the fluid and cultured in monolayer in medium consisting of 20% Chang medium D (Irvine Scientific, Irvine, CA, USA), MEM-alpha GlutaMAX (Life Technologies, Carlsbad, CA, USA), 15% embryonic stem cell-qualified fetal bovine serum (Life Technologies, Carlsbad, CA, USA) and 100 µg/mL Normocin (InvivoGen, San Diego, CA, USA), then cryopreserved at passage 2. Following thawing, cells were incubated at 37 ℃, 5% CO_2_, 95% humidity until 80% confluent. We detached cells from the plate using Accutase (Thermo Scientific, Waltham, MA, USA) for further passages or experiments. For the design of this study, AFMSCs from 3 separate batches of amniotic fluid were combined in cell culture.

### Animal protocol

All animal protocols were approved by the New York University School of Medicine Institutional Animal Care and Use Committee. We obtained male and female diabetic (Lepr^db/db^) mice, aged 6–8 weeks, from The Jackson Laboratory (Bar Harbor, ME, USA). All Lepr^db/db^ mice used in the experiments had blood glucose concentrations of > 400 mg/dL. We obtained C57BL/6N wild type (WT) mice from Taconic Biosciences (Rensselaer, NY, USA). We housed these mice in a temperature-controlled, virus-free barrier animal facility with a 12-h light/12-h dark cycle and maintained them on chow diet and water ad libitum.

### Preclinical wounding model

Following sedation with 2% isoflurane, we used the foot pad pinch test to confirm that each mouse was properly anesthetized. We used an established murine model of excisional wound healing [28]. Briefly, we first removed hair on the mouse dorsum using a hair trimmer and Nair (Church & Dwight, Princeton, NJ, USA). We created paired 10-mm full-thickness wounds extending through the panniculus carnosus on the dorsum of the mice using a punch biopsy tool, and then splinted the wounds open with 0.6 mm thick silicone stents of 10 mm inner diameter and 20 mm outer diameter (silicone sheets from Grace Bio-Labs, Bend, OR, USA). Once positioned over the wound, we secured the stent to the full thickness of the dorsal skin with interrupted 4–0 braided sutures (Henry Schein, Inc., Melville, NY, USA). We placed the sutures to ensure that they do not disrupt the boundary of the wound. We then covered the stented wounds with occlusive dressing with a 10 mm window to allow air exchange, while preventing changes in wound dimensions due to gnawing or pulling from the mice. Analgesics provided for 3 consecutive days post-operation aid in preventing mice from disrupting the wound healing. We used standardized photographs at regular intervals post-op to photometrically analyze the percent wound remaining, calibrating against the internal diameter of the 10 mm silicone stent to correct for magnification, perspective, or parallax effects. We recorded time to wound closure as the number of days for complete scab detachment and resemblance to intact skin by gross visual inspection. We calculated percent wound remaining [(unhealed wound area)/(original wound area)] using digital measurements of wound photographs (Adobe Systems, San Jose, CA, USA). We used area under the curve (GraphPad software) to assess the wound burden [29]. Wound photographing, calculation of unhealed wound areas and wound burden using photographs were all blinded.

### AFMSC administration

At passage 5, we detached cells using Accutase, then centrifuged at 300 × *g* and resuspended in AFMSC media at 5 × 10^4^ cells/µL. Using a sterile micropipette, we administered 10 µL of AFMSC suspension (5 × 10^5^ cells total) directly into the wound, immediately after excision and stenting. For cell tracing studies, we incubated the cells with 1,1′-Dioctadecyl-3,3,3′,3′-Tetramethylindocarbocyanine Perchlorate (DiI, D3911, Invitrogen) at a final concentration of 4 ng/µL. Following a 2 h incubation, we washed cells 3 times with fresh, warmed 1 × PBS prior to detachment. Before detaching cells, we confirmed and photographed DiI labeling using a Cytation 5 (Biotek, VT, USA). The treatment administration was blinded, as well as subsequent monitoring and wound analysis to prevent any subjective biases.

### In vivo AFMSC imaging

Prior to AFMSC administration, we positioned anesthetized mice in an In Vivo Imaging System (IVIS) Lumina III (Perkin Elmer, MA, USA) and captured fluorescence at excitation 560 nm and emission 610 nm. We re-imaged the wounds on the mice immediately following administration of DiI-labeled AFMSCs. We continued imaging at 6, 12, 24 and 48 h post-administration and analyzed the data using Living Image software (Perkin Elmer, MA, USA). Mice were identified with numbers and treatments were blinded.

### Wound tissue harvest

For histological analyses, we excised the wound with a 5 mm margin diameter, dissected along the cranial-caudal axis and fixed the skin wound tissue in 4% paraformaldehyde overnight at 4 °C. For wounds treated with DiI-labeled AFMSCs, following overnight fixation to preserve any fluorescent signal, we cryopreserved the tissue using 15% and 30% sucrose sequentially, then embedded in optimal cutting temperature medium (Neg 50, FisherScientific, CA, USA). For those treated with non-labeled AFMSCs, wound tissue samples underwent routine histologic processing for embedding in paraffin and sectioning at 5 µm thickness. We used deparaffinized skin-tissue sections for hematoxylin and eosin staining, performed by the Experimental Pathology Laboratory at NYU School of Medicine.

### DiI-label detection in wound tissue sections

We dried slides with 20 µm frozen wound tissue sections for 60 min at room temperature, prior to rehydration in 1 × PBS and 2 further washes. We counterstained the sections using Hoechst 33342 and mounted with Prolong Gold Antifade Mountant (ThermoFisher Scientific, CA, USA). We analyzed and photographed sections on a Nikon Ti2E (Nikon, NY, USA).

### Immunostaining

After deparaffinization of wound tissue sections, we performed heat-mediated antigen retrieval in a pH 6 sodium citrate buffer (Dako). Then we blocked and permeabilized using a 5% normal donkey serum (017-000-121, Jackson ImmunoResearch) and 0.01% Triton-X (9002-93-1, Sigma Aldrich) in PBS/0.2% bovine serum albumin/0.75% glycine. We immunostained with primary antibodies for rabbit anti-mouse Arginase 1 (16,001-1-AP, Proteintech) and rat anti-mouse F4/80 (ab6640, Abcam), and donkey anti-rabbit and donkey anti-rat IgG secondary antibodies, conjugated to Alexafluor 594 and Alexafluor 488, respectively. We counterstained the sections using Hoechst 33342 and mounted with Prolong Gold Antifade Mountant (ThermoFisher Scientific, CA, USA). We analyzed and photographed sections on a Nikon Ti2E (Nikon, NY, USA).

### RNA isolation and quantitative PCR

We lysed AFMSCs on the culture plates using Trizol, per the manufacturer’s guidelines (ThermoFisher Scientific, CA, USA). For wound tissues, we homogenized the tissue in Trizol as well, using ceramic beads in a Beadmill for 2 cycles of 30 s 6 m/s runs (ThermoFisher Scientific, CA, USA). We added 200 µL chloroform per 1 mL Trizol, agitated the samples and allowed phase separation for 5 min at room temperature. Following centrifugation at 12,000 × *g* for 15 min at 4 °C, we removed the aqueous phase containing RNA and precipitated using isopropanol. We loaded the precipitated RNA onto Qiagen RNeasy columns and continued using the manufacturer’s instructions, incorporating the on-column DNA digest. Final RNA was eluted in a 32 µL volume of RNAse-free water. We quantified total RNA using a Nanodrop and reverse transcribed 500 ng RNA using High-Capacity cDNA Reverse Transcription kit (ThermoFisher Scientific, CA, USA). We used SYBR Green PCR Master Mix (ThermoFisher Scientific, CA, USA) to detect and quantify transcripts. Primer sequences are available upon request.

### Statistical analysis

Data is represented as mean ± standard deviation of at least 3 separate biological replicates. Utilizing Graphpad Prism, we used a Student’s *t*-test when comparing 2 sets of data and Dunnet’s procedure for 3 or more sets with a fixed control, with statistical significance level at p < 0.05. We determined that for an 80% powered analysis with an effect size of 5, we required minimum 4 diabetic wounds per type of treatment for the time to closure studies.

## Results

### Single AFMSC dose alters diabetic wound healing time

To determine efficacy of AFMSCs in promoting wound closure, we used a validated humanized excisional wound model in adult Lepr^db/db^ type 2 diabetic mice [9, 28, 30]. Excisional wounds in Lepr^db/db^ mice demonstrate severe wound healing delays compared to their syngeneic counterparts [9, 30], but also respond to therapeutic interventions, making them a broadly accepted pre-clinical model [28, 31]. In order to stimulate the events resembling human cutaneous wound healing, a silicone stent was sutured to the entire thickness of the skin, including the panniculus carnosus muscular tissue. This tissue is only present in mouse skin, unlike humans, and stenting allows for formation of granulation tissue without premature contraction of the panniculus carnosus.

**Fig. 1:**
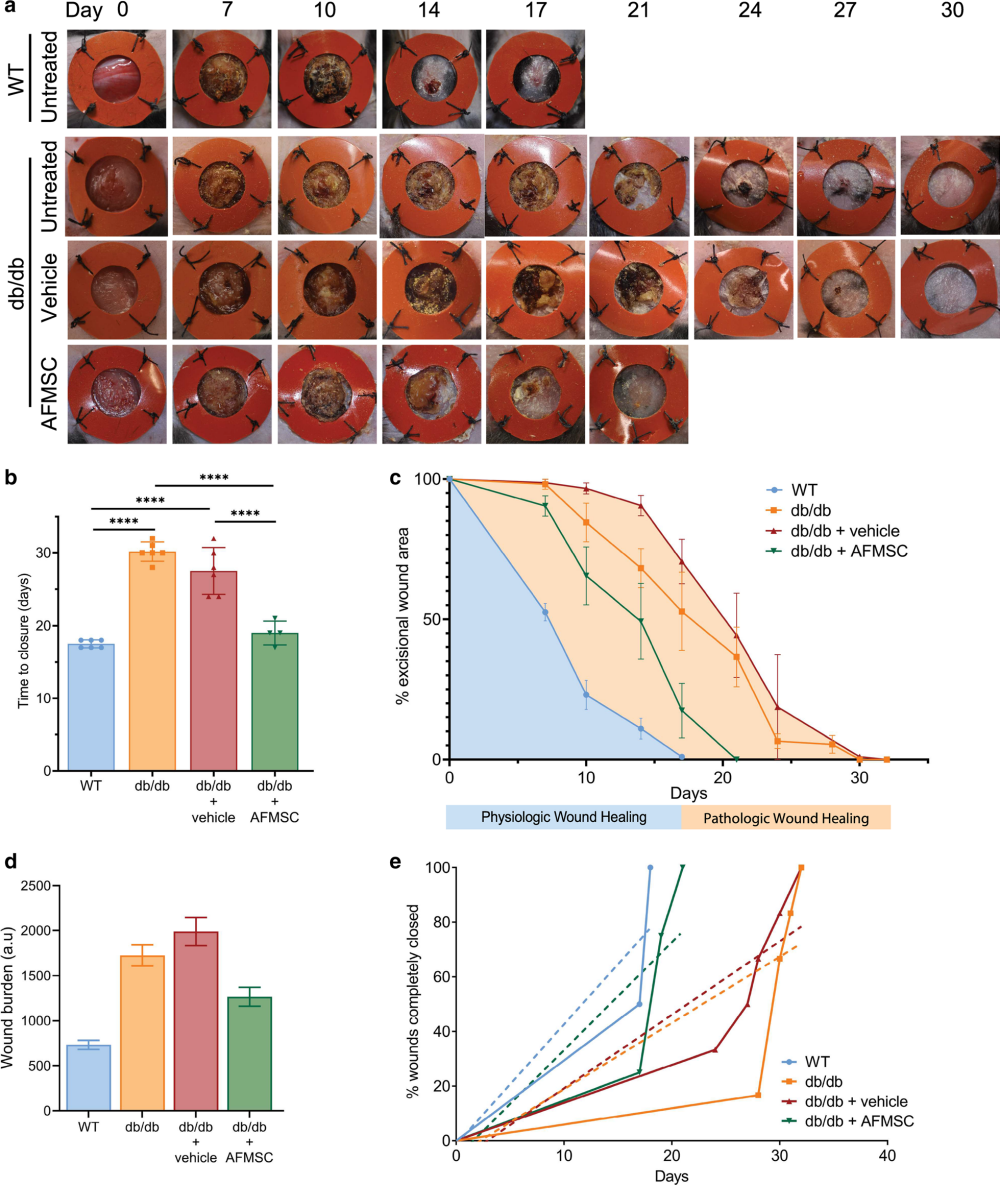
Topical application of AFMSCs accelerates diabetic wound healing. **a** Photographs of WT and diabetic wounds at indicated time points with indicated treatments. **b** Mean time to wound closure. ****p < 0.001, n ≥ 3. **c** Percentage remaining wound area over time. Shaded area under the curve represents wound burden. **d** Wound burden, integrated area under the curve in **c**, in arbitrary units (a.u). **e** Wound closure rate. Dashed regression lines fit to each treatment group

We administered a single dose of 5 × 10^5^ cells to each wound immediately following excision and stenting. We monitored the wound surface area, observing for loss of eschar and appearance of re-epithelialized skin. Photometric recording demonstrated that AFMSCs significantly reduce time to wound closure to 19 ± 0.82 days compared to vehicle (cell culture media) treated diabetic wounds at 27.5 ± 1.30 days (p < 0.0001) or untreated diabetic wounds at 30.17 ± 0.54 days, p < 0.0001 (Fig. 1a, b). AFMSC treatment results in a 75% decrease in time to closure compared to vehicle-treated and an 80.3% decrease compared to untreated diabetic wounds. The wound closure times indicate that the AFMSCs, rather than their culture media, produce the acceleration in diabetic wound healing. We did not observe a significant difference between vehicle-treated and untreated diabetic wounds. Importantly, AFMSC treatment pushes the wound closure trajectory of diabetic wounds towards that of WT wounds (Fig. 1c). Analysis of integrated area under the curve in Fig. 1c, of remaining wound areas plotted against time, reveals the wound burden which is an indicator of wound healing capacity [32, 33]. AFMSC treatment reduces the wound burden of diabetic wounds, improving the chances of a favorable faster healing trajectory (Fig. 1d). We calculated a 57.7% and 46.5% decrease in wound burden of AFMSC-treated diabetic wounds, in contrast to vehicle-treated and untreated wounds, respectively. Linear regression analysis of the wound healing times per wound and type of treatment provides the rate of healing (Fig. 1e). Comparison of the slopes confirms the wound burden data, that AFMSC treatment promotes a faster healing rate than vehicle-treated or untreated diabetic wounds (Fig. 1e). Our results show that early passage AFMSCs have the capacity of promoting and normalizing diabetic wound closure.

### AFMSCs cannot be detected long term and do not cause adverse reactions upon administration

**Fig. 2:**
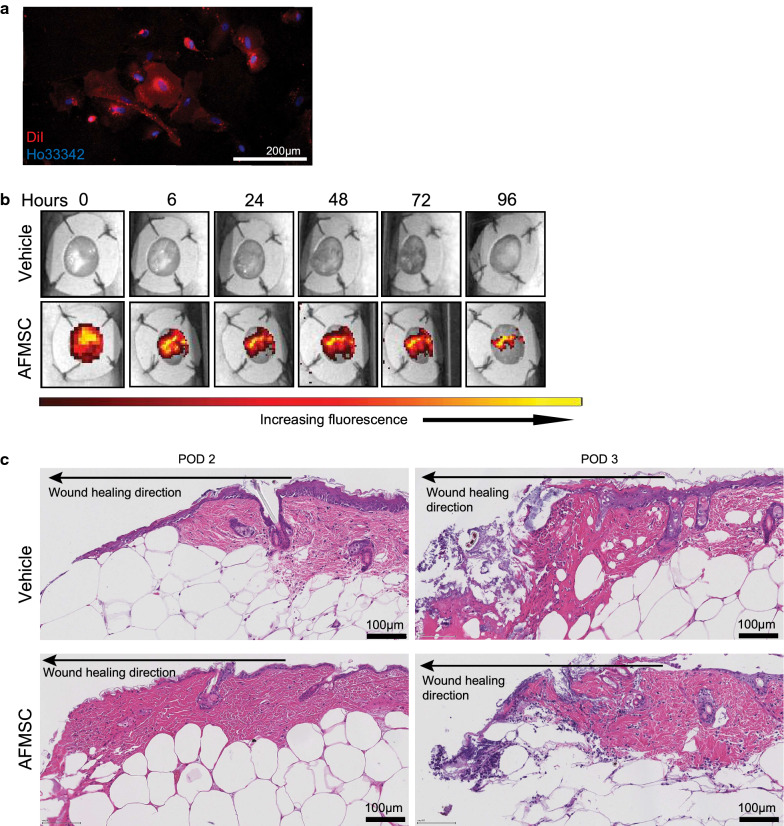
AFMSCs do not induce adverse effects in mouse diabetic wounds. **a** DiI-stained AFMSCs in culture. **b** IVIS-generated images of diabetic wounds following topical application of DiI stained AFMSCs, until signal could no longer be detected. Images are representative of n ≥ 3. **c** Representative H&E image of diabetic wound tissue at post-operative days 2, and 3 after vehicle treatment and AFMSC treatment, n ≥ 3. Scale bar 100 µm

Based on the success of AFMSCs in driving wound closure, we wanted to determine the length of time that AFMSCs can be detected in the diabetic wound following topical AFMSC administration. We labeled AFMSCs while still in culture with DiI, immediately prior to wound application. DiI is a lipophilic dye and we confirmed by fluorescence microscopy that AFMSCs cell membranes were labeled with the dye in our chosen conditions (Fig. 2a). Following topical administration to the diabetic wounds, we monitored the DiI fluorescence detection at regular intervals until loss of signal. Only AFMSC and not the cell culture media produced fluorescence. We detected DiI epi-fluorescence from day 0 immediately following application until 72 h later (Fig. 2b). The eschar already forming by this time point contributed to some autofluorescence (still visible at 96 h post-administration). We did not detect any DiI fluorescence in 20um diabetic wound tissue sections at post-op days 1, 2 or 3 (data not shown).

We also wanted to determine whether AFMSCs trigger an immunogenic response in diabetic wounds, as the source is xenogenic. To this end, we analyzed H&E stains of diabetic wound tissue sections from post-op days 2 and 3. AFMSCs did not cause any additional cellular influx at the time points we analyzed, compared to vehicle-treatment (Fig. 2c). Our results thus far suggest that AFMSCs do not engraft or orchestrate additional inflammatory influx in diabetic wounds.

### Administration of AFMSCs alters wound histology

**Fig. 3:**
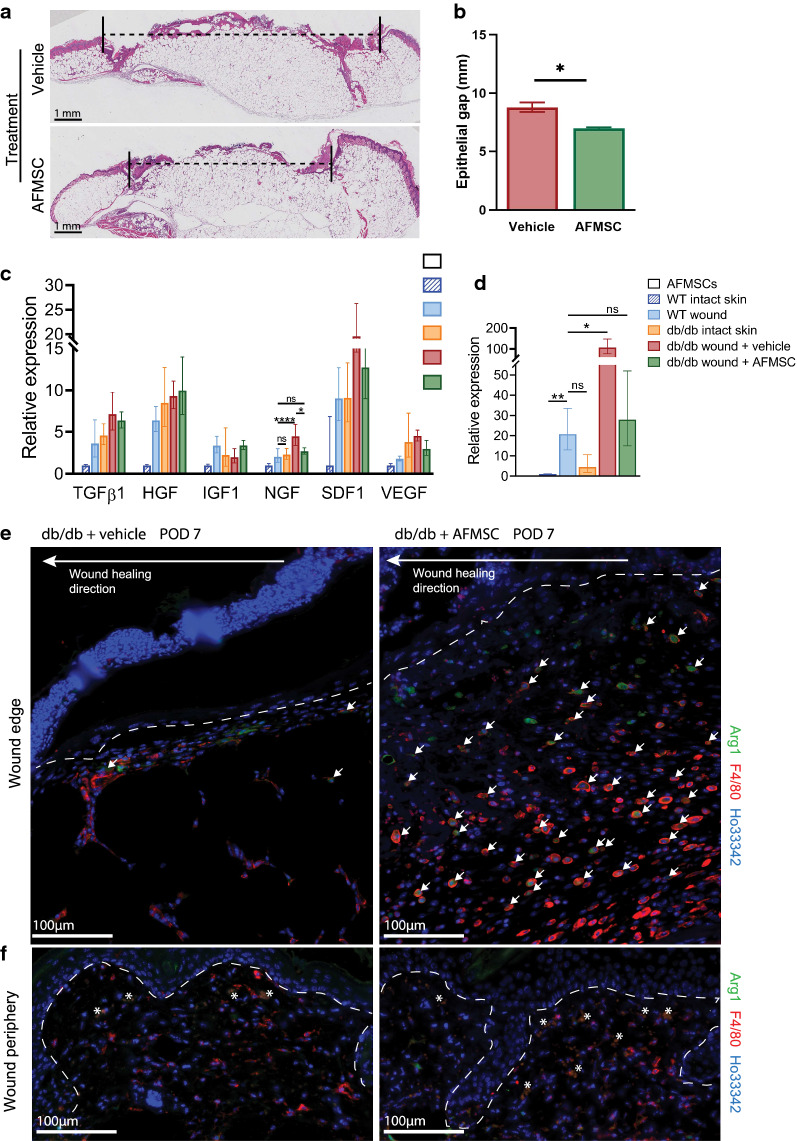
AFMSC treatment induces molecular change in diabetic wound bed. **a** Representative H&E tissue sections of diabetic mouse wounds from post-op day 7. Black dashed line indicates the epithelial gap. *p < 0.05. **b** Quantification of epithelial gaps, n ≥ 3. **c** Relative gene expression of wound healing factors, with mouse models and treatments as indicated at post-op day 7. *p < 0.05. ****p < 0.0001. ns, not significant, n ≥ 3. **d** Relative gene expression of IL6. *p < 0.05. **p < 0.01, ns, not significant, n ≥ 3. **e** Representative images of immunodetection of F4/80 and Arg1 expressing cells in diabetic wound tissues with treatments as indicated. Tissues are counterstained with Ho33342. White arrows, F4/80^+^/Arg1^+^ cells. Asterisk, autofluorescence from erythrocytes

To further analyze the impact of AFMSC administration on cutaneous tissue repair and regeneration, we analyzed the extent of re-epithelialization based on the epithelial gap, the distance spanning the wound epidermal leading edges. Diabetic wound tissue sections collected at post-op day 7 showed that AFMSC-treated wounds had significantly reduced epithelial gap with a mean of 6.88 ± 0.05 mm compared to that of vehicle-treated wounds with a mean of 8.9 ± 0.24 mm, p < 0.05 (Fig. 3a, b).

We also investigated changes in gene transcripts in diabetic wounds following treatments. Gene expression of NGF, a wound repair associated growth factor, has higher baseline expression in intact diabetic skin (Fig. 3c). In fact, the expression levels are similar to that of C57 wounded skin. Upon wounding, NGF expression significantly increases in diabetic wounds, but the AFMSC treatment normalizes the gene expressions towards that of WT wounds. We did not find any significant changes in expression of TGFβ, HGF, IGF1, SDF1 or VEGF transcripts in whole wound bed tissues, though expression trends are apparent. All these factors increase in expression after wounding in WT mice, but the expression in unwounded diabetic skin is already elevated, suggesting stalled wound healing in the latter. Interestingly, AFMSCs do not express the transcripts for any of the genes analyzed, indicating that any modulations observed in the wound tissue are not due to transcriptional programs within AFMSCs. Our results indicate that the gene expression changes are induced in the wound bed cells, potentially through trophic mechanisms or cellular induction, by administration of AFMSCs.

We detected significant changes in expression of the cytokine interleukin 6 (IL6) (Fig. 3d). The unwounded or intact Lepr^db/db^ skin demonstrates significantly higher IL6 expression compared to WT intact skin, but on par with wounded WT tissues. WT wounds show a significant upregulation of IL6 in post-op day 7 wounds (p = 0.0056) in contrast to WT intact skin. However, IL6 expression is upregulated greater than 4-fold in vehicle-treated diabetic wounds, compared to WT wounds (p = 0.0195). AFMSC treatment reduces the IL6 expression in diabetic wounds such that it is no longer significantly different compared to that of a WT wound.

As IL-6 is associated with change of macrophage phenotypes [34] we analyzed macrophage populations in the wound tissue. As early as post-op day 7, arginase1 (Arg1) expression, which indicates polarization of macrophages towards a pro-repair phenotype [35], colocalized with F4/80 + macrophages in both AFMSC-treated and vehicle-treated wound tissue sections. However, the larger granulation tissue area in AFMSC-treated diabetic wounds demonstrated significantly higher numbers of Arg1 + /F4/80 + cells, in contrast to vehicle-treated wounds (Fig. 3e). Comparison of immunostaining on wound peripheral skin tissue sections demonstrated that similar numbers of F4/80 + macrophages are present regardless of vehicle or AFMSC treatment (Fig. 3f). Our results suggest a change in local macrophage polarization signals in response to AFMSC administration.

## Discussion

A search for “mesenchymal stem cells” on clinicaltrials.gov reports more than 1100 registered human clinical trials using these cells for therapeutic purposes [36]. Evidence points towards multipotent stromal cells or MSCs having extensive remedial potential and being especially promising for tissue regeneration. MSCs, by definition, express cluster of differentiation (CD) markers including CD73, CD90, and CD105, which relates their potential to differentiate into tissues of multiple lineages [17]. Adding to this, these cells have also been found to have immunomodulatory properties [17]. These findings have led to their recognition as possible agents for mediating and enhancing the healing process, especially in environments such as diabetic ulcerations, which have abnormal immune regulation [37]. Extensive work has been done to establish both the safety and functionality of these MSCs for tissue healing. Safety studies have found that they do not result in any serious adverse effects, particularly because they have low immunogenicity [38]. With regard to functionality, studies using systemic exogenous MSCs have shown them to selectively migrate to sites of injuries, where they interact with signaling molecules [39] and also secrete factors involved in orchestrating repair [40]. With that being said, MSCs used in clinical trials are mainly derived from bone marrow (BM) and adipose tissue. However, the non-invasively sourced AFMSCs may be a better candidate for clinical applications. They also compare favorably to BM-derived MSCs because of their lower expression of MHC class I [40] and to adipose-derived ones because of their greater secretion of angiogenic factors [26, 40]. Even more, amniotic fluid is easily obtained during caesarean section deliveries, and according to the latest CDC report, in 2018 alone there were over 1 million caesarean births [41]. MSCs derived from amniotic fluid have easy expansion protocols and are capable of more than 300 population doublings [13, 14, 42]. Yet, there are currently only around six clinical trials utilizing AFMSCs, none of which are for diabetic wound repair [36].

The treatment of diabetic ulcers is a unique challenge given that these wounds have multifactorial etiology. The healing process is impeded by stunted cell differentiation and proliferation, deficient extracellular matrix formation, reduced growth factor release, diminished neovascularization, and persistent expression of pro-inflammatory cytokines [43]. Effective therapies should facilitate complete, non-delayed wound closure and prevent reoccurrence. In the present study, we demonstrate that AFMSC therapy can ameliorate diabetic wounds by significantly decreasing the total time to wound closure. Untreated Lepr^db/db^ murine diabetic wounds take longer to heal, however, once the AFMSCs are applied, the healing time closely resembles that of WT wounds, suggesting that the typical wound healing sequence is restored (Fig. 1). As the Lepr^db/db^ mice used for this study have compromised signaling at wound sites [27], we can deduce that the allogeneic AFMSCs modulate wound healing based on their paracrine competence. Our results showing efficacy of AFMSCs corroborates previous findings in studies using rat [25] and diabetic NOD/SCID mice models [40]. We used a non-invasive AFMSC delivery strategy and applied cells to the wound microenvironment of the Lepr^db/db^ diabetic mouse skin, emulating application of the therapy immediately after debridement to expose surrounding healthy tissue. This allows for maximum contact with the wound bed cells, while eluding invasiveness. These findings showing the success of AFMSCs in treating humanized wounds in diabetic mice leads us to believe that this therapy is feasible for translational medicine.

For further clarity of therapeutic events, we analyzed the diabetic wound microenvironment post-treatment. Intriguingly, histological and molecular changes in AFMSC-treated wounds most closely related wound healing events in wild type mouse wounds. We investigated the presence and differentiation of macrophages, which are multifunctional and dynamic in the wound milieu. In the early phase, pro-inflammatory macrophages are activated due to the innate immune response [44]. In the later phases of wound healing, macrophages transition to an anti-inflammatory or pro-healing population which are involved in reestablishing integrity of the skin [35]. Their role includes support of revascularization, formation and restructuring of granulation tissue, collagen deposition and maturation, and re-epithelialization [35, 45]. Notably, macrophage plasticity is lost in chronically inflamed diabetic tissues, which have a higher presence of pro-inflammatory macrophages [35]. We found noteworthy modulation of IL6 expression in the diabetic wound bed as an outcome of AFMSC treatment (Fig. 3). Unwounded diabetic skin demonstrated similar IL6 transcripts as that in wounded WT skin, and AFMSC therapy successfully reduced IL6 expression of diabetic wounds to the same range as that of WT wounds. IL6 is typically recognized as a pro-inflammatory cytokine, which agrees with our findings that diabetic skin presents a hyper-inflammatory baseline, which is further upregulated upon wounding, and neutralized by AFMSC therapy. Interestingly, recent studies have suggested that IL6 could foster pro-healing macrophage polarization dependent on the microenvironment [34]. Our findings indicate that at least by 7 days after excision and therapy, AFMSCs affect macrophage differentiation to foster the transition to the later phases of healing. This offers a favorable perspective for the clinical use of AFMSCs in the treatment of diabetic ulcers.

Besides efficacy, we also examined safety of treating diabetic ulcers with AFMSCs. We did not detect any evidence of an immunogenic response in AFMSC treated mice compared to the control groups. We did not detect long term presence of AFMSCs in the mouse wound bed either. Based on our method of DiI signal detection, the loss of signal could indicate clearance of AFMSCs from the topical administration site or that the numbers of AFMSCs remaining are too low for detection by epi-fluorescence or tissue sections. The eschar that forms on the wounds can impede epi-fluorescent signal detection or contribute to false signals. Our findings reflect the consensus in peer-reviewed literature that MSCs and stromal cell types used for promoting repair have low to virtually no engraftment, unless in bone tissues [5, 46]. We therefore anticipate that clinical use of this therapy should have a decreased risk of engraftment syndrome, pending clinical trial confirmation of this in long term studies.

It is important to recognize that further investigation of AFMSC secretome and paracrine activity would contribute to a better understanding of their regenerative potential. In particular, it is necessary to decipher the mechanisms by which AFMSCs normalize the macrophage populations in the diabetic wound environment. Successful clinical resolution is further dependent on whether AFMSC is effective in treating wounds of different sizes based on dosage titration. Moreover, for proficient clinical use, AFMSC manufacturing needs to be optimized. We anticipate that given the lack of immunogenicity of AFMSCs, banked cells from multiple batches will significantly increase clinical availability. We were limited in our ability to assess the safety of a scaled up AFMSC supply because we used cells from a small donor population. Nevertheless, the outcome of this study supports that AFMSCs therapy may be revolutionary for the treatment of non-healing diabetic wounds.

## Conclusion

Our study demonstrates that AFMSCs show promise for promoting cutaneous wound healing in pre-clinical models of delayed wound healing. Topical and limited therapy with AFMSCs can resolve the stalled wound repair in mouse type II diabetic wounds and drive transition towards closure. AFMSCs induce molecular and cellular change in the diabetic wound microenvironment to effectively reduce time to healing of full-thickness wounds, offering a feasible translational route for the therapy of chronic wounds in patients with diabetes.

## Data Availability

All raw data used and/or analyzed in this study are available from the corresponding author on request.

## Abbreviations

AFMSC: Amniotic fluid multipotent stromal cells
Arg1: Arginase 1
db/db: Lepr^db/db^ type II diabetic mouse model
DiI: 1,1′-Dioctadecyl-3,3,3′,3′-tetramethylindocarbocyanine perchlorate
H&E: Hematoxylin and eosin
HGF: Hepatocyte growth factor
Ho33342: Hoechst dye 33342
IGF1: Insulin-like growth factor 1
IL6: Interleukin 6
NGF: Nerve growth factor
POD: Post-operative day
SDF1: Stromal derived factor
TGFβ: Transforming growth factor β
VEGF: Vascular endothelial growth factor
WT: Wild type, C57 mice
